# From Presbyopia to Cataracts: A Critical Review on Dysfunctional Lens Syndrome

**DOI:** 10.1155/2018/4318405

**Published:** 2018-06-27

**Authors:** Joaquín Fernández, Manuel Rodríguez-Vallejo, Javier Martínez, Ana Tauste, David P. Piñero

**Affiliations:** ^1^Department of Ophthalmology (Qvision), Vithas Virgen del Mar Hospital, 04120 Almería, Spain; ^2^Department of Ophthalmology, Torrecárdenas Hospital Complex, 04009 Almería, Spain; ^3^Department of Optics, Pharmacology and Anatomy, University of Alicante, Alicante, Spain; ^4^Department of Ophthalmology (OFTALMAR), Vithas Medimar International Hospital, Alicante, Spain

## Abstract

Dysfunctional lens syndrome (DLS) is a term coined to describe the natural aging changes in the crystalline lens. Different alterations in the refractive properties and transparency of the lens are produced during the development of presbyopia and cataract, such as changes in internal high order aberrations or an increase in ocular forward scattering, with a potentially significant impact on clinical measures, including visual acuity and contrast sensitivity. Objective technologies have emerged to solve the limits of current methods for the grading of the lens aging, which have been linked to the DLS term. However, there is still not a gold standard or evidence-based clinical guidelines around these new technologies despite multiple research studies have correlated their results with conventional methods such as visual acuity or the lens opacification system (LOCS), with more scientific background around the ocular scattering index (OSI) and Scheimpflug densitometry. In either case, DLS is not a new evidence-based concept that leads to new knowledge about crystalline lens aging but it is a nomenclature change of two existing terms, presbyopia and cataracts. Therefore, this term should be used with caution in the scientific peer-reviewed literature.

## 1. Introduction

Dysfunctional lens syndrome (DLS) describes the natural lens changes in the crystalline lens and has been helpful in educating patients, staff, and doctors about these changes for years [1]. The crystalline lens aging from presbyopia to cataracts is coined in a single term which includes three stages. The stage 1 has been popularly suggested [1, 2] from 42 to 50 years old and corresponds to the term of presbyopia, when accommodation has been lost but light scatter remains relatively limited. In the stages 2 (50 to 65 years old) and 3 (65 or older), the ocular scatter increases and a lens replacement-based procedure may be warranted [1, 2]. These ranges of age used in clinical practice can be questionable since there is broad agreement among different authors that from the age of 30–40 years there is typically a slow drift towards hyperopia [3]. Presbyopia can, therefore, start before the age of forty and the light can scatter after sixty [4]. The DLS, therefore, is not a new evidence-based concept that leads to new knowledge about crystalline lens aging, but it is a nomenclature change of two existing terms, presbyopia and cataracts. It results from the new treatments and diagnosis tools in the modern refractive cataract patients [5]. The aim of the creation of this concept was to facilitate the comprehension of lens aging for patients, and it has sense as nowadays the treatment of Stage 1 (presbyopia) is based on a combination of guidelines for refractive error [6] and cataract [7] treatment. Therefore, it seems coherent to talk about DLS in the adult eye-preferred practice pattern instead of cataract if also presbyopia treatment is considered [7]. The main aim of the current article is to review the current evidence around the new diagnostic tools that might lead to a future change of paradigm linked to the DLS term that may serve to improve the decision criteria for the current alternatives to the treatment of presbyopia and cataracts.

## 2. Aging of the Crystalline Lens

### 2.1. Physiology of the Lens

Different alterations in the refractive properties and transparency of the lens are produced during the development of presbyopia and cataract. Although it is not well understood how lens cellular structure and function initiate changes in refraction and transparency, a common underlying mechanism in the pathology of cortical and nuclear cataract can be attributed to the failure of the microcirculation system to regulate cell volume in the lens cortex, or deliver antioxidants [8], such as the Glutathione [9, 10], to the lens nucleus [11]. Donaldson et al. [11] rigorously described the physiological optics of the crystalline lens and the development of cataract, suggesting future possible treatments based on functionality changes at the cellular level. Therefore, we recommend reading the work of Donaldson et al. [11] for a better knowledge of the physiology of the lens aging and these potential promising future treatments.

### 2.2. Internal Aberrations

The internal aberration variations with age have led to some controversies for years as was pointed out by Smith et al. [12]. It is agreed that the relaxed lens has a negative spherical aberration (SA) value close to the positive value of the corneal surface [12], exhibiting then a balanced compensation up to around 45 years of age [13]. Alió et al. [14] and Amano et al. [15] reported an increase with age of coma and positive SA attributed to the crystalline lens. The negative spherical aberration of the lens can be partly explained by the inherent Gradient of Refractive Index (GRIN) [16–19], and the decrease of internal negative SA with age by the increase of the refractive index of the plateau (nuclear) region of the lens in old people that has a size reaching a maximum value at 60 years old [20]. These results are in agreement with Sachdev et al. [21] and Rocha et al. [22] who evaluated the level of high order aberrations (HOAs) in eyes with cortical and nuclear cataracts. On the contrast, Lee et al. [23, 24], Wu et al. [25], and Faria-Correia et al. [26], conversely to previous authors, reported that negative internal SA was increased in nuclear cataract as well as Kuroda et al. [27] and Zhu et al. [28] who also suggested that the opposite happens in cortical cataracts, with an increase of the positive total SA measured with Hartman–Sack aberrometers. This was attributed to the fact that wavefront in the central pupillary area relatively delays in nuclear cataracts and relatively advances in normal subjects and cortical cataract [27]. The hypothesis surrounding these findings are opposite to the GRIN changes with age, but the authors explain this by a major increase of refractive index in the nucleus comparing to the surrounding which means that for nuclear cataracts, the plateau tendency might be not presented.

### 2.3. Scattering

Light is scattered when enters into the eye due to optical imperfections or lack of transparency from the optical media and is the main cause of glare perception [29]. The scattering should not be confused with optical aberrations, while optical aberrations deflect light in small angles (<1°), light scatter produces straylight over large angles (>1°) [30]. There are two methods for the assessment of light scattering, the light scattered into the retina (forward scattering) or the light scattered backward (backward-scattering). The slit-lamp evaluation of lens opacities is based on backward-scattering; however, it is important to note that backward-scattering represents light that not reaches the retina, and the light that reaches the retina cannot be derived from this backward-scattering [31]. First, studies assessing backward-scattering which implied the human lens were published in the mid-seventies [32] and were aimed at characterizing the molecular changes associated with the early stages of cataractogenesis [33]. A clinical device based on this measure was developed in 2008 and the term dynamic light scattering was coined for referring to the measurement of scattering due to light-particle interaction as a function of time [34, 35]. However, dynamic light scattering is focused on measuring changes in molecules, such as *α*-crystallin [36], whose decrease has been related to the risk of developing cataract instead of understanding the implication of the scattering on the visual function.

### 2.4. The Impact of Age-Related Optical Changes on Visual Performance

Besides the numerical increase of internal aberrations or scattering, the clinical significance of these parameters is determined by their influence on the visual performance. Despite internal aberrations are increased with age, it is important to note that this increase does not necessarily have a clinically significant influence on visual performance as there are also changes occurring in pupil size with age [37]. Thus, even though there is a variation of spherical aberration with age, this variation does not deteriorate visual performance in eyes with small pupils [38]. Furthermore, neural changes in the aging visual pathways, in agreement for *P* pathways and controversial for *M* [39], can have a role on the decrease on visual performance, but the role seems to be less relevant when compared against the influence of the optical properties of the ageing eye [40]. Then, it is reasonable to conduct estimations of the affected visual performance with objective systems despite not considering the neural processing. Moreover, the prediction of the possible visual performance achievable after surgery is not possible until a clinical system evaluating the visual acuity through the cataract is developed [41], without the limitations of past technologies that have not demonstrated a clinically useful prediction of postoperative best-corrected visual acuity, such as the potential acuity meter and the visometer [42].

The gold standard for measuring the visual performance in clinical practice is the high contrast visual acuity (VA). Internal HOAs increase with age is related with a decrease of VA [38]. However, an increase of aberrations generating visual complains is not always related to a high contrast photopic VA deterioration [21]. Similarly, the increase in scattering has shown poor although statistically significant correlation with VA [30]. Therefore, VA provides an incomplete assessment of visual performance and other clinical tests, such as contrast sensitivity or straylight, should be added in the clinical evaluation of cataract [43]. In fact, despite the VA still remain as the gold standard for driving license (0.3 logMAR in Europe) [44], some researchers have claimed to include other metrics which have demonstrated a higher risk to be involved in car accidents, such as contrast sensitivity with Pelli Robson test (1.25 log cut-off value) [45, 46] and straylight (1.4 log cut-off value) [45], or motion sensitivity and mesopic high-contrast VA for driving at night [47].

The crystalline lens aging has a different impact factor on contrast sensitivity function (CSF) depending on the level of scattering or HOAs. Although both affect to CSF, Zhao et al. [48] reported that the loss in CSF when both scattering and HOAs are present cannot be explained as a summation of the single impact factor of scattering or HOAs. Indeed, less reduction can be obtained when combining scattering and HOAs than the impact factor of each one separately [48]. This suggests that there is a compensatory neural processing, with different impact for different spatial frequencies. While single analysis of HOAs has a higher impact on higher spatial frequencies, scattering has a more significant impact on middle spatial frequencies.

## 3. Objective Technologies for Lens Evaluation

Objective technologies for grading the development of cataract are based on the measure of these previous variables, internal aberrations and scattering. These technologies include densitometry measured with Scheimpflug camera devices or anterior segment optical coherence tomography (AS-OCT), internal wavefront aberrations obtained from the subtraction of the corneal from the total wavefront aberrations, and the direct measure of the point spread function with a double-pass system.

### 3.1. Densitometry

The objective lens densitometry (OLD) is measured by Scheimpflug camera-based devices. The Pentacam HR (Oculus Optikgeräte GmbH, Wetzlar, Germany) includes the pentacam nucleus staging (PNS) classification which evaluates the mean densitometry in a continuous scale from 0 to 100 [49] or in an ordinal classification from 0 to 5 [50]. The software automatically detects the nucleus location and measures the densitometry in a cylindrical three-dimensional template. A limitation of the software is that cortical cataracts can produce shadowing artefacts or misplacement of the reference template that may affect to the PNS [49]. Studies have found that the analysis of the nuclear region, as the PNS performs, has a higher correlation with visual performance than the average of the whole lens [51]. The average lens density at the nucleus location is correlated with VA (*r*=0.44 [52], *r*=0.63 [53], *r*=0.76 [51]) as well as with contrast sensitivity for four spatial frequencies (*r*=−0.30 at 3 cpd, *r*=−0.55 at 6 cpd, *r*=−0.60 at 12 cpd, and *r*=−0.48 at 18 cpd) [51]. AS-OCT has been also proposed recently for grading the density of the lens with the purpose of predicting phacoemulsification energy; however, subjective grading through Lens Opacification System III (LOCS III) has resulted in higher correlations with the phacoemulsification energy than AS-OCT or Scheimpflug [54]. The latter is in controversy with the report by Faria-Correia et al. [49]. In any case, the grading of presbyopia and cataracts through AS-OCT seems to be a promising technology, not only because of the OLD measure, but also due to the possibility to measure the dynamic changes of the crystalline lens during accommodation [55].

### 3.2. Wavefront Aberrometers

Nowadays, several devices subtract the corneal wavefront derived from corneal topography to the total wavefront directly obtained from raytracing or Hartman–Shack aberrometry. These devices include Irx3 (Hartmann–Shack; Imagine Eyes, Orsay, France), KR-1W (Hartmann–Shack, Topcon, Japan), Keratron (Hartmann–Shack; Optikon, Rome Italy), iTrace (ray-tracing; Tracey Technologies, Houston, TX), and OPD-Scan (Automated Retinoscopy; Nidek, Gamagori, Japan). In the early development of the measurement of internal aberrations, some caution was pointed out because of the lack of reliability of obtaining these from aberrometry and corneal topography (CT) [56]. The main problem of these devices was the two-step measurement that required a perfect realignment during topography and later measure of the wavefront [57]. In fact, internal aberration comparison between devices has led to significant differences [58], in some cases in a considerable degree [59]. However, it is also important to note that devices based on different technologies such as KR-1W or iTrace have reported similar results to describe an increase of negative internal spherical aberration in nuclear cataract [24–26, 28]. Based on the measurement of internal aberrations provided, some aberrometers such as the iTrace have developed an index that ranks overall lens performance from 0 (very poor) to 10 (excellent) points. This index has shown correlations with VA, (*r*=−0.67 [49], *r*=−0.70 [53]) but as far as we know, there are no studies showing the correlations of this index with other metrics such as contrast sensitivity.

### 3.3. Double-Pass System

The objective scatter index (OSI) comes from the double-pass technique that examines the forward-scattered light, which causes degradation of retinal images in eyes with cataract [60]. Unlike wavefront technologies, the double-pass technique also considers the scattered light; therefore, modulation transfer function in early stage cataract can be better related with visual function than the optical quality characterized using data from wavefront devices [61, 62]. OSI has been used to classify cataracts in normal (<1.0), early (from 1.0 to 2.9), mature (from 3.0 to 6.9), and severe (≥7.0) [60]. Control groups without cataract rarely shows an OSI value higher than 1.0, although some cases in control groups [63] or young subjects [64] can result in slight values over 1.0. A recent study has demonstrated that OSI has sensitivity and specificity values to discriminate healthy and cataractous eyes of 89% and 100%, respectively, when a cut-off value of 1.18 is used [4]. The mean cut-off value for early cataract differs among authors. While Artal et al. [60] reported a value around 2.0 for early cataract, Galliot et al. [65] classify early cataract with a mean OSI value of 3.7. The criteria for cataract classification should not be confused with the criteria for surgery. The cut-off value for which a cataract is recommended to be operated in a sample of subjects with decimal VA > 0.6 was set by Paz et al. [66] at 2.1 considering two groups of subjects for which surgery was previously recommended or not according to conventional ophthalmological criteria (area under the receiver operating characteristic curve of 0.83). Zhang and Wang [67] also suggested conducting capsulotomy in patients operated on with cataract surgery when an OSI value of 3 was measured. Interestingly, they reported 5 cases of patients with subjective symptoms and VAs better than 0.15 logMAR but with OSI values above 3. In these cases, VAs remained constant after capsulotomy but with a decrease of OSI below 1.3 and with the resolution of symptoms.

The OSI has been also compared with LOCS III classification scale in nuclear (NC), cortical (CC), and posterior subcapsular (PSC) cataracts. Although there exists consensus about a clear correlation between OSI and LOCS III in NC, this is not as clear in PSC or CC [68, 69]. LOCS III is not always correlated with OSI because the central pupil area (4 mm) is not always covered by some types of cataracts [63]. An opacification can be detected on slit-lamp examination, but without induction of visual impairment in some cases. Indeed, Paz Filgueira et al. [68] reported that LOCS III in PSC was not correlated with psychophysical parameters, such as visual acuity, contrast sensitivity, and the straylight parameter (log(s)). Likewise, Vilaseca et al. [63] found a greater dispersion of OSI and VA in eyes with PSC and CC.

The correlations of OSI and VA have been widely studied. Paz Filgueira et al. [68] reported nonsignificant linear correlations between OSI and VA, but these authors found a significant correlation between OSI and straylight parameter (log(s)). Cochener et al. [70] reported a correlation of OSI and VA (*ρ* = 0.48, *P* < 0.001) similar to that reported by Crnej et al. [71] (*r*=0.45). In contrast, Pan et al. [52] reported a correlation between OSI and VA of *r*=0.78. Cabot et al. [69] reported that this correlation varied among nuclear (*ρ* = 0.7), cortical (*ρ* = 0.5), and posterior subcapsular (*ρ* = 0.6) cataracts. Similarly, Martínez-Roda et al. [4] also reported an OSI-VA correlation dependency on the type of cataract (*r*=−0.40 nuclear, *r*=−0.38 cortical, and *r*=−0.48 posterior subcapsular). Besides VA, correlations of OSI with contrast sensitivity have been also reported for nuclear cataract (*r*=−0.56 at 3 cpd, *r*=−0.45 at 6 cpd, *r*=−0.39 at 12 cpd, and *r*=−0.40 at 18 cpd), but these have been shown to increase for posterior subcapsular cataract, as happened with VA [51]. It is especially interesting the study of Vilaseca et al. [63] who reported an exponential decay model with correlations of *r*=0.88, *r*=0.84, and *r*=0.84 for nuclear, cortical, and posterior subcapsular cataracts, respectively. The authors also remarked that in some cases, a dense cataract can drastically increase the OSI and therefore the intraocular scattering, whereas its impact on VA is not as strong.

The evaluation of the OSI has been also reported two months after cataract surgery with monofocal IOL implantation in eyes with a preoperative mean OSI around 11.5, showing significant differences between spherical (3.2 ± 0.8) and aspheric lenses (2.5 ± 0.8) that were not detected by means of visual acuity [72]. Park et al. [73] reported that only subjects above 70s resulted in a significant lower OSI after implantation of a monofocal IOL in comparison to nonimplanted subjects. However, Park et al. [73] reported a mean OSI in pseudophakic eyes of 2.21, and other authors have reported lower values, such as Jiménez et al. [74] who found a decrease in the mean OSI from 7.44 preoperatively to 1.48 at three months, or Lee et al. [75] who reported a mean OSI of 1.38 in pseudophakic eyes and Chen et al. [76] who found a mean OSI of 1.45 and 2.50 in eyes implanted with monofocal and multifocal IOLs, respectively. Lee et al. [77] also reported a mean OSI of 1.82 with multifocal IOLs. However, the validity of all these studies with MIOLs is questionable because of the known limitations of the near-infrared optical performance of diffractive multifocal intraocular lenses [78], or the first-pass in the double-pass technique that can be affected by the size of the first ring [79]. Finally, OSI has found to be correlated with straylight parameters and even related to driving safety [43]. Martínez-Roda et al. [44] estimated that an OSI of approximately 3 may be considered as a safe margin for driving.

### 3.4. Decision Criteria with Objective Systems

Decisions about crystalline lens surgery are based on a benefit/risk balance. Risks can be related to intraoperative or postoperative complications. Major complications are potentially sight-threatening and include infectious endophthalmitis (0.02%–0.05%) [80, 81], anterior segment syndrome [7], intraoperative suprachoroidal haemorrhage (0.46%) [82], cystoid macular edema (1.17%) [83], retinal detachment (0.03%) [64], persistent corneal edema, IOL dislocation, ptosis, corneal decompensation, diplopia, and blindness [7]. Other adverse events can be presented during the surgery, such as anterior capsular tears (0.55%–0.79%) [84, 85], posterior capsule tears or rupture with or without vitreous loss (1.8%–3.5%) [86, 87], or during the postoperative period, such as iritis (1.53%) [84], corneal edema (0.53%) [84], and posterior capsule opacification (4.2%) [87]. Considering that these complications have decreased with years [81, 82, 88], it is important to note that decisions should be taken considering the most recent evidence and also considering factors associated with the increase of incidence of complications, such as ocular comorbidities [87], age [89], sex [81], and combined surgery [81, 88].

Although new metrics have demonstrated superiority in the diagnosis of cataract [90], the common primary indicator for cataract surgery is still preoperative VA [91]. Kessel et al. [92] reported that there is a lack of scientific evidence supporting the use of preoperative VA to guide the clinician in the decision of recommending surgery or not. However, VA is effective for regulating the number of required surgeries in order to prevent an unmet population [93]. In Spain, VA of 0.4 decimal is considered the cut-off value for surgery indication in most of public hospitals, but this criterion is the result of the need for attending population within the possibilities of the health resources and therefore, the criteria can change depending on the possibilities of each country and the aging population with the potential of developing cataract [93]. Kessel et al. [94] also pointed out that evidence-based guidelines can change practice patterns unless they are counteracted by the reimbursement system. Therefore, the cut-off value of 0.4 decimal used in public hospitals in Spain is more an economic point to prioritize surgeries in an ageing population than a risk-benefit balance based on health parameters. In fact, a cut-off value of VA is not recommended by the American Academy of Ophthalmology guidelines that recommends instead surgery when the visual function no longer meets the patients' needs [7].

If the preoperative VA is not a good indicator to guide the clinician in the recommendation of cataract surgery with monofocal IOLs, the recommendation of implantation of multifocal IOLs is even a more complicated process. The contraindication criteria for MIOLs have been well established [90], but the stage of DLS at which the patient will achieve a highest satisfaction after a MIOL implantation still remains unclear. Satisfaction after cataract surgery with MIOL implantation might be associated with nonvisual variables such as expectations considering the previous use of spectacles [95, 96] and quality of care given during the hospital stay [97]. The desire for achieving spectacle independence in a wide range of distances remains the most important issue for MIOLs indication, but since satisfaction with multifocal IOLs will vary due to nonrelated vision factors [90, 98, 99], other variables such as personality should be also considered [90, 100].

The DLS criteria suggest that refractive lens exchange (RLE) should be considered as a treatment alternative in Stage 2 when ocular scatter increases [1, 2] and some authors have suggested also in Stage 1 in subjects who have presbyopia and reduced visual quality under low light conditions, high hyperopia [101], or high myopia [6]. However, it is also important to note the risk factor associated to high hyperopia, such as choroidal effusion and macular edema [102], or in high myopia, such as the percentage of retinal detachment which is around 2–8% of eyes [6], and the risk factors associated to this detachment [102].

Besides risks of surgery, there are other vision-related adverse events due to MIOL implantation that can influence on patients' satisfaction. Adverse events of MIOLs include reduced contrast sensitivity, halos around point sources of light, multiple or ghosting images, and glare [7]. Halos and glare, also known as dysphotopsias, are intrinsically associated to the monofocal [103] or multifocal IOL technologies [98, 99, 104], resulting in one of the most important complains [99, 103]. However, despite being intrinsically associated to the technology, not all the patients refer disturbances associated to dysphotopsias, probably because these phenomena are only perceived under certain conditions, such as driving at night looking at a bright light source against a dark background [104] due to neural adaptation [105] or due to patients' personality [100]. On the contrast, it is also important to note that some adverse effects produced by MIOLs, such as glare or contrast sensitivity reduction, also appear during cataract development. Thus, it is reasonable to expect that a patient with levels of contrast sensitivity, visual acuity, or dysphotopsia at far distance equal or better than those presented preoperatively will be more satisfied with a MIOL because a loss in visual quality at far would be not perceived as disturbing while an improvement at intermediate and near vision without spectacle would be perceived. Furthermore, it is important to note that a loss in contrast sensitivity is expected after MIOL implantation due to the lens split light in more than a focus, but this loss of energy at far distance will not be linearly correlated with the reduction in contrast sensitivity mainly due to optical quality in the normal eye is 10 times better than the capability of the neural system to process contrast [106]. Then, a reduction of energy of 50% will not correspond to a decrease of contrast sensitivity of 50%.

A wide knowledge of the visual quality at the preoperative stage and the achievable at the postoperative stage will lead to a benefit/risk based on vision-related adverse effects of MIOLs in addition to the benefit/risk based on surgery adverse events. In terms of dysphotopsia, Puell et al. [107] reported that halo radius started to increase exponentially from the age of 50 during cataract development. In this study, authors excluded cataracts below level 2 of LOCS III with independence of the age and they reported a maximum mean radius of 160 arcmin from 70 to 79 years. In another study, Palomo-Álvarez et al. [91] included subjects with cataract above level 2 of LOCS III obtaining a mean radius of 2.40 log arcmin (251 arcmin). The mean halo radii with monofocal IOL was 190 arcmin and with *a* + 3.00 D multifocal IOL was 225 arcmin [108]. Considering the values reported by these previous studies, we can consider that a cataract grade 2 is necessary to avoid exceeding the halo radii of the preoperative stage after cataract surgery with implantation either of monofocal or multifocal IOLs.

Our research group also conducted the same analysis for contrast sensitivity and visual acuity after MIOLs implantation (Table 1). The mean monocular contrast sensitivity and VA achieved with different MIOLs was close to those reported for grade 1 cataract by Martínez-Roda et al. [4] and the OSI was around 1.5. Therefore, considering the published literature, a cataract grade 1 would result in similar far contrast sensitivity than that achieved with a MIOL. However, the dysphotopsia associated to *a* + 3.0 D add bifocal IOL would be greater, being necessary a grade 2 cataract for obtaining a similar halo size. This conclusion should be interpreted with caution because it was obtained with different studies including different samples, and future paired design studies should be conducted including preoperative and postoperative halo ring size or contrast sensitivity in the same subjects.

## 4. Conclusions

The term dysfunctional lens syndrome (DLS) is commonly used in congresses instead of referring to presbyopia or cataracts [1, 2, 5, 101]. However, few research papers [49, 53, 113] use this term in studies linked with new objective technologies for grading the cataract development. The term DLS can be criticized by professionals and researchers arguing that this term was born with technology and not from evidence [114], even though some authors claimed that the term has been used for over 15 years [1]. In this review, we evaluated the current evidence around these new technologies in order to help the modern surgeon to take decisions about cataract and refractive lens exchange procedures based on these devices. However, there is still few studies addressing cut-off values recommended for implanting a monofocal or a multifocal IOL. Likewise, studies including benefits from surgery in patients measured preoperatively and postoperatively are required. Considering the limitations of these devices to measure optical quality after the implantation of a multifocal lens, the only mode to conduct this task is relating the preoperative objective with subjective measures of the visual performance, such as contrast sensitivity, and estimating the cut-off value based on their association with objective measures in the preoperative visit. In this sense, we can conclude according to literature that a preoperative OSI of 1.5 may be considered as a value equivalent to the visual performance achieved by the patient after the implantation of a MIOL as for this OSI value preoperative and postoperative contrast sensitivity are similar. However, this conclusion should be interpreted with caution because it is achieved by means of evaluating the results obtained from different studies, and future paired studies including information of the same eye during the preoperative and postoperative visit are required. Considering the current state of limited evidence on the potential usefulness of new technologies to characterize clinically age-related optical changes and the lack of a gold standard or clinical guidelines, the use of the term DLS should be used with caution in the scientific literature, being preferable the use of the terms presbyopia and cataract. A new terminology only should replace the previous one when this offer new evidence-based information not covered by the previous one.

## Figures and Tables

**Table 1:** Monocular visual performance and optical quality in cataract and eyes implanted with multifocal intraocular lenses.

	CDVA	3 cpd	6 cpd	12 cpd	18 cpd	OSI
*Control* (52 to 65 years) [4]	−0.10	1.69	1.92	1.51	0.93	0.67
LOCS III (grade 1)	0.03	1.56	1.81	1.41	0.99	1.56
LOCS III (grade 2)	0.18	1.52	1.70	1.29	0.81	3.47
LOCS III (grade 3)	0.31	1.43	1.57	1.12	0.80	5.88
LOCS III (grade 4)	0.59	1.31	1.30	0.90	0.46	10.23
*Multifocal*						
Restor + 2.5 [109]	0.01	1.49	1.64	1.31	0.90	—
Restor + 3.0 [109]	0.01	1.51	1.65	1.22	1.07	—
Fine vision [109]	0.01	1.6	1.70	1.26	0.94	—
Tecnis symfony [110]	−0.04	1.6	1.69	1.31	0.89	—
Restor + 3.0 [111]	−0.13	1.73	1.93	1.56	1.12	—
Restor + 4.0 [111]	−0.13	1.70	1.92	1.54	1.09	—
Tecnis + 4.0 [112]	−0.03	1.86	1.99	1.68	1.21	—
*Mean*	−0.04	1.63	1.77	1.39	1.02	—
